# A Monograph on Ovarian Tumors

**Published:** 1857-06

**Authors:** T. M. Tweed

**Affiliations:** North Liberty, Ohio


### SELECTIONS.

*A Monograph on Ovarian Tumors; with an extended view of Ovariotomy as a means of cure.* By T. M. Tweed, M. D., of North Liberty, Ohio.

[CONCLUDED.]

The Radical Cure of Cystic Dropsy by Ovariotomy.—Ovariotomy is apparently an easy, although a very dangerous operation. Nothing can be more simple than making an incision from the ensiform cartilage to the pubis; it is done at any *post mortem* examination; but the effects of such an opening in the living subject, are fearful in the extreme, and in many instances, fatal.

In the present state of our knowledge, opening the peritoneal cavity is no longer considered to be an operation of necessity fatal, and it does not require a number of experiments, like those of Dr. Blundell's, to put the question at rest. Nearly three hundred cases of ovariotomy are already recorded, and the results of these cases, it will be our duty to investigate.

Before we admit the legitimacy of an operation like that under consideration, we must distinctly determine three questions, which are of the utmost importance, and to these we shall now direct our attention.

I. *What is the common course of ovarian dropsy, and what the result of its ordinary treatment?*

II. *What are the results of those cases in which the cyst has been extracted?*

III. *And what is the fair conclusion to be deduced as to the practicability of the operation, on a review of the two former questions?*

Before entering upon these questions, it will be proper to be thoroughly acquainted with the opinions held by those who are worthy of our confidence. We do not wish to place much stress upon individual opinion, and especially where it has not been formed by actual experience; but we do hold that the opinions of our predecessors, and those who have obtained eminence in their profession, are worthy of some consideration and reflection.

The operation of the extraction of cystic tumors is only of recent date in England, though practiced successfully in the United States nearly fifty years ago. It is slowly, but surely, taking its position among the acknowledged legitimate operations of Surgery.

It is said to have been proposed first by Vanderhaar, and afterward by Delporte, Moraud, and Logger. It was first undertaken by L'Amonier of Rouen, and afterward, in 1809, successfully performed by Dr. McDowel, of Kentucky. The operation was performed at Edinburgh, by Mr. Lizars, in 1823, who was unable to find a tumor; and in 1826, Dr. Granville of London, operated on a patient but was unable to remove the tumor. Since that time, about three hundred patients have been operated on with variable success. About one in four of all the known operations have proved fatal.

Mr, Clay and Frederick Bird, of England, have operated oftener than any other two men in the world.

Prof. Atlee, of Philadelphia, has operated oftener than any other American surgeon. Next to him in the number and success of his cases, was the late Dr. Buckner, of Cincinnati.

Sir Charles Bell thought the dangers arising from the operation itself, were quite sufficient to deter the surgeon from undertaking it.

It is the opinion of Dr. W. Hunter, that incision ought not to be attempted; he says, "It has been proposed indeed by modern surgeons, deservedly of the first reputation, to attempt a radical cure by incision and suppuration, and by the excision of the cyst. I am of the opinion that excision can hardly be attempted, and that incision and suppuration will be found by experience, to be an operation which cannot be recommended but under very peculir c rcumstances."

Mr. Lawrence thinks extirpation of the ovarium is an operation so likely to kill the patient, that he does not think it advisable to proceed to it.

In the opinion of Liston, these operations are exceedingly unjustifiable.

Prof. Meigs even goes so far as to say, that "operations for the extirpation of the diseased ovary, are not to be justified by the most fortunate issue in any ratio of cases."

And, Dr. Seymore, who has written on diseases of the ovaria, states that the arguments against such an operation are numerons and strong, and the probabilities of success, very small. "If the tumor be not large, or the woman's health unbroken, she may live many years—as long as is allowed to humanity, in the enjoyment of a tolerable existence. If the health be much broken, the cure of so large a wound, in a weakened constitution, would be difficult, if not in the majority of cases, impossible."

"If connected with scirrhus in other parts of the body, it is inadmissable; and if the growth itself be of the nature of fungus hœrmatodes, all experience tells us that, should the patient survive or the wound heal, the disease will recur in other vital organs of the body. Nor do its difficulties rest here; when these growths enlarge to a great extent, they must frequently adhere, *and then the operation is out of the question.* If all these exceptions then are estimated, the case which remains, in which such risk is advisable, and such an operation feasible, with any fair chance of a happy result, is rare indeed."

These, then, are some of the opinions held by men high in the profession, upon the subject of ovariotomy. The authorities which favor it, will be considered, when treating of the operation itself. We will, so far as we have the ability, produce every fact within our knowledge, both for and against the radical treatment by extirpation, and endeavor to form a just estimate of its practical value. In a matter so momentous to the interests of humanity, and of such transcendent importance to science, any other course would be criminal.

I. *What is the ordinary course of ovarian dropsy, and what the result of its usual treatment?*

This question has been examined to some extent in a preceding part of this paper. But we must now descend to particulars. As before stated, this disease is most common in, and usually attacks patients about, the prime of life, when the sexual organs are fully developed, and their functions in a state of excitement. When once established, it continues to increase, and in half the cases, terminates in death in two years. Ninety, out of one hundred and twenty-three, died in four years, leaving only thirty-three patients, or not quite one in four, to survive that period.

In order to prevent this severe mortality, the means already referred to have been introduced into practice, and we have seen with how little success, even as palliative measures. We have shown that paracentesis has the preference as a palliative; that is, the lives of some patients have been more prolonged by the operation than by any other treatment. It now becomes us to inquire, whether or not this is the *usual* result.

It is admitted by all, that this operation "is the beginning of the end;" that it will require repetition during longer or shorter periods; exhaustion or inflammation, produced by the operation itself, generally terminating the case.

In unilocular cysts, this operation may be repeated several times, without producing serious results; and while the disease retains this character, it may be relieved for many years by the operation; but experience demonstrates that the tendency of these unilocular cysts is to produce others upon their inner surface, and when this is effected, the danger of the operation is greatly increased.

There can be no doubt that, as in the cases mentioned, tapping does prolong for a considerable period the lives of some patients; but all will agree that, after the first tapping, the necessity of the operation becomes much more frequent, the re-accumulation of fluid being more rapid, until the patient is worn out by disease.

In order to ascertain the mortality following this operation, and the extent of the benefit derived from it, we have collected a table of forty-six cases in which the results were recorded. Of these 46 patients, 37 died, and 9 recovered, and are supposed to be living. Of the 37 patients, more than half died within *four* months; 27 of the 37, died within one year; and of this number, 18 were only tapped once. This exhibits a great mortality, not only to the operation generally, but to the first tapping in particular. Admitting the nine cases reported to have recovered, were all perfect cures, we have more than five deaths to one recovery; and it is more than probable, that most of those nine ultimately died of the disease.

It may be said, that in these cases, the disease itself would have proved fatal, had not paracentesis been resorted to. In many, no doubt this remark holds good, and some would have fallen a sacrifice; but when considered as a means of cure, these data furnish a pretty just estimate of the value of tapping.

We are aware that our conclusions do not correspond with those of many practitioners. The old notion of the harmless nature of paracentesis, and the perfect safety of its frequent repetition, is an incorrect one; and referring to individual practice we find, that for one of the extraordinary cases that survive fifty or sixty tappings, many die, and that in a very short period.

At the very best, then, tapping is a very dangerous means of palliation; when had recourse to it must be frequently repeated; and the relief afforded between each operation, becomes gradually less, and the dangers consequently, greater. These are reasons—strong and persuasive—for seeking some other mode of treating ovarian dropsy, that will promise more hopeful results.

II. *What are the results of those cases, in which the cyst had been extirpated?*

Dr. Blundell, nearly fifteen years ago, instituted a number of experiments to show that the peritoneum might be opened with comparative safety, and stated it to be his opinion, that abdominal surgery would be better understood, and that the extraction of the ovary would become a legitimate operation. Gastrotomy, since that time, has been performed more than two hundred times, and the results are before the profession in the form of tabular statements. The statistical researches of Lee, Churchill, Atlee, and Buckner have been published, and a reconstruction of the Tables is unnecessary; the more so, as we are assured, that the ratio of mortality attending the operation has steadily diminished, with the multiplication of operations.

But even here we must be cautious in our judgment. While we may rest assured, that we have all the successful cases, are we quite certain that we have a full report of those which have proved unsuccessful?

To this inquiry we regret that we are compelled to give a negative answer. Mr. Philips states, "I have heard particulars of other *five* cases, of which at least *three* were unsuccessful; but I cannot venture to use them. As any honorable man should be equally ready to publish his unsuccessful cases, we may look for the authentic particulars hereafter."

This opinion is confirmed by Dr. F. Bird, who says, "It is deeply to be regretted that the profession were unable to form any correct opinion on the subject [Ovariotomy] from motives that could not be too strongly censured. Unsuccessful operations had been most carefully suppressed, while those in which a happier termination occurred, had been hurried into publicity, even before the patient had been fully recovered, and while the ligature was still contained in the abdominal cavity. Within the last few weeks, the abdomen of a patient had been laid open, in which no tumor was contained; in another example in which the operation had been performed, death had been ushered in with all the symptoms of strangulated intestine; in another, in which the abdominal section had been employed, the patient had quickly died; yet were all these cases carefully concealed, while those in which recovery had taken place, were made the subject of daily advertisement."

The writer of this Essay is aware of three cases, all of which proved fatal shortly after the operation, but of which no authentic particulars have as yet been published. Let the scorn and contempt of all honorable men, rest upon such shameful and criminal concealment! As long as such a selfish spirit as this actuates medical men, so long must we hope in vain to arrive at a final or correct judgment; for the deaths not recorded, would make a material difference in the ratio of mortality.

So complete is Dr. Atlee's analysis of the statistics of gastrotomy, that it would be a work of supererogation to review the whole ground occupied by his labors. It is sufficient for our purpose to state that the statistics indicate a rate of mortality equal to twenty-five per cent. This result, of course, includes all the exploratory operations; for they were commenced with the view of completing them, had circumstances justified it. They involved the section of the peritoneum, and the consequent hazard of the complete operation.

What strikes us as very remarkable in the statistics, is the great number of cases in which, from an error of diagnosis, the surgeon has been compelled, after the operation was commenced, to abandon it. And this is one of the most serious arguments against the operation. Lizars, Clay, Walne, Dieffenbach, West, and other distinguished surgeons, have committed this grave error. Indeed, the tables show, that two out of every eight patients submit to the abdominal section, when the tumor can not be removed on account of adhesions, or when in fact no tumor is to be found.

It has been stated that ovariotomy exhibits a less degree of mortality, than most of the acknowledged capital operations. If this be true, it is a valuable argument in favor of the procedure.

Malgaigne has shown that out of 852 amputations of the extremities of all kinds (including those of the fingers and toes), which were performed in the Parisian Hospitals, from 1826 to 1841, 332 died, or about four out of every ten proved fatal.

Among these, out of 201 amputations of the thigh, 126 died, or six in every ten; out of 192 amputations of the leg, 106 died, or five and a half in every ten; out of 91 amputations of the arm, 41 died, or four and a half in every ten. Of the amputations of the thigh, in 46 cases the operation was performed for severe injury of the limb; of these 34 died, or more than seven out of every ten.

At the Glasgow Infirmary, the mortality in cases of amputation, is four in every ten; and at the Edingburg Infirmary, five in every ten.

Mr. Philips has collected the histories of 171 cases in which the large arteries were tied; of these, 57 died, or about three and a half in every ten. Sir A. Cooper, in his work on Hernia, records 36 deaths in 77 operations for that disease, or nearly five in every ten These are a few of the recorded results of some of the capital operations, and we find that the ratio of mortality in them, is even greater than in ovariotomy. But, perhaps, this is not a fair comparison, because in the operations named, there is a necessity compelling the surgeon to act immediately (in many cases) whatever may be the co-existing circumstances; whereas, in cases of ovarian disease, no such necessity exists. The health of the patient may be good; many live a considerable period—on an average, four years; but if the operation is fatal, it is so in a few hours, or days at most.

In all the numerous cases recorded as having undergone the abdominal section—without a removal of the cyst, the cause of failure has been the want of a correct diagnosis, either as to the nature of the tumor (some being omental and others uterine), or the extent and complication of the adhesions.

It is of the utmost importance, that a correct opinion be formed, as to the nature and extent of the adhesions which may connect the cyst with the peritoneum or the abdominal viscera. The question hence arises, are there any means by which this complication can be ascertained?

If we study the experience of the past, I fear we shall come to the conclusion, that this complication can not always be accurately ascertained before the operation; for surgeons the most cautious and skillful have been deceived, and adhesions found in cases where they have been least suspected.

There are some presumptive signs, which, after a careful examination, may become very valuable.

When an ovarian sac has attained a size, which is productive of great inconvenience and distress to the neighboring organs, the parietes of the abdomen become greatly attenuated, and the space between the recti abdominalis is much enlarged; this is well seen if the patient be told while lying on her back, to raise herself into the sitting posture, without the aid of her arms; and if the sac within be free in its motions, it will be protruded through the space between the recti muscles, and present an oval enlargement. But supposing the cyst to be intimately adherent in front, no such bulging will take place. We have tested this sign in several instances, and remarked the truthfulness of it.

Another indication of this sort is valuable, and that is the action of the diaphragm upon the tumor. If the measurment of the abdomen be obtained after the patient has taken a deep inspiration, and again, after a full expiration, we will find that the two measurments frequently vary an inch or two, *when the cyst is free;* showing that the diaphragm, in the inspiratory movement, had driven down the unattached cyst, while the expiratory effort allowed it to repossess its original position in the abdomen.

Again, by placing the hand upon the abdomen, and with the fingers grasping the parietes, we may frequently feel that the movements of the the cyst are unconnected with those of the abdominal walls; and this is much more marked when an ascetic fluid is present; here, also, a sudden tap will allow the fingers to come in contact with a hard substance below, proving that a space exists between the tumor and walls. When ascites is present, the sac is usually free from adhesions.

The position of the pelvic viscera gives great assistance to the elucidation of the diagnosis.

If the bladder is free, and after being emptied of urine, is filled with air by means of an electric tube, it will pass above the pubis, and can be detected by the resonant sound it produces on percussion; thus proving that no adhesions in that position prevent its ascent. The uterus, if perfectly free, can be thrown by the uterine sound upon the rectum, or to either side, showing that the cyst is connected with it. Another test which has been resorted to, with the view to detect adhesions to the pelvic viscera, is to place the patient on her knees with the shoulders lower than the pelvis; in this position, the tumor is thrown by gravity into the abdomen, and toward the diaphragm, and by examination per rectum and vagina, we may almost certainly determine whether adhesions exist.

If no adhesion exist, the tumor will have receded and left all the pelvic viscera free. But if they do exist, either to the rectum, uterus, or bladder, the weight of the tumor will drag with it the organs attached, which may readily be determined by the finger; and if adherent to the peritoneum lining to pelvic cavity, it is recognized by the fact, that we are unable to raise the tumor above the brim of the pelvis, by pressure made through either the rectum or vagina.

Another important test of adhesions, is tapping. If there be any doubt of this complication, before the operation of extraction is recommended, the patient should be tapped a few weeks previous to the operation; by this means we are enabled to ascertain whether adhesions exist or not, especially if present in the anterior parietes of the abdomen. On the withdrawal of the fluid, the walls of the abdomen have a drawn and puckered appearance, and are observed to follow closely the contracting cyst, while the cyst itself does not descend into the pelvis. This is observed when adhesions are present. Whereas, when the cyst is free from adhesions, it may be found after the evacuation, low in the pelvis, forming a hard tumor, while the walls of the abdomen remain free. If now the tumor is grasped through the flabby and relaxed walls of the abdomen, and lifted up from the pelvis, we have pretty conclusive evidence that no adhesions exist, either to the walls of the abdomen or pelvic viscera. After we have obtained this information; the cyst may be allowed to refill, and its extraction proposed.

A fine example of this character came under the observation of Dr. Buckner; and by the means already described, he detected adhesions and solid matter. He remarks, "The patient was very desirous to undergo the operation of ovariotomy, but fearing extensive adhesions I tapped her, and drew off thirteen gallons of fluid of albuminous character. The walls of the abdomen were distinctly seen to follow the con racting cyst. A solid mass which was present proved that the cyst could not from the adhesions, pass down in the pelvis, for it remained stationary on both sides, and above the umbilicus; the operation was therefore abandoned. She was tapped several times subsequently, but finally died; and a post-mortem examination revealed extensive adhesions, and fully proved the correctness of our diagnosis, and the propriety of decling the operation of extirpation."

Adhesions are supposed to exist, if the "crepitus" pointed out by Dr. Bright is present. But they may exist and no such sensation be produced. In some cases this sound is very distinct, while in others it is not so, and therefore it cannot be relied on.

It has been thought that the history of the case, would throw some light upon the existence of adhesion; that they might be traced to some inflammatory attack, and that after such an attack, they were to be apprehended.

Mr. Philips says, in an admirable article on the subject, that "the crepitating sign pointed out by Dr. Bright, is only present when the adhesions were recent; and as to the motion of the tumor with the diaphragm, considerable adhesions may exist, without much interfering with it. Our main reliance therefore upon the signs of peritonitis; if the evidence is clear that peritoneal inflammation had existed, it is probable that adhesions were present, but we may find adhesions where there has been no reason to suspect peritonitis. Still extensive adhesions, in the absence of symptoms of peritonitis, are by no means common. It is then mainly upon this point, that we must rely before proceeding to the operation.

The size of the cyst, and consequent pressure on the surrounding parts, cannot account for adhesions being present, since we know that ovarian sacs of equal size or greater magnitude may exist, although no such morbid connections are formed.

The mobility of the tumor was considered indicative of the non-existence of adhesions. This is always a very favorable sign, and shows that the tumor is not closely connected with the surrounding tissues; but extensive adhesions may exist even in this state. Dr. Clay, in his second case, met with this difficulty; he found the tumor quite movable in all directions, except for a few inches anteriorly, where he supposed it to be adherent; but on making the incision for the operation, the tumor was found to be adherent in all directions.

From these observations we find, that all the usual signs by which we endeavor to discover the existence of adhesions, are not always to be relied on. If the tumor protrudes between the divided recti, it does not indicate the absence of adhesions *posteriorly*, although it may be depended on as far as the anterior surface is concerned.

And last of all, even the mobility of the tumor is not to be depended on, for it may be readily pushed from one side of the abdomen to the other, and yet there may exist adhesions so strong, that they require a scalpel for their division.

But although the dependence on these symptoms singly, may lead us into error, the combination of many of them, will generally be conclusive. Supposing the patient, when rising by her own exertions, protrudes the cyst as an oval, bulging tumor through the space left by the separation of the recti; that on a deep inspiration, the tumor is pressed downward toward the pelvic cavity, and then recedes on an expiration; that the bladder is free, and can ascend into the anterior part of the abdomen, when filled with air; that the uterus and rectum are found free, and can be moved at pleasure by the finger or uterine sound; that all crepitation is absent, and the tumor tolerably movable; then we may with satisfaction say that adhesions do not exist. An additional evidence would be, if the patient had previously been tapped, the entire disappearance of the sac after the operation.

Some state, that after tapping the sac usually adheres to the puncture of the parietes of the abdomen; but this is not correct. It may occur, and no doubt does, in some instances, but not generally.

Besides the complications of adhesions, another important fact ought to be borne in mind in the treatment of these tumors,: viz, the frequent concurrence of other organic diseases. A reference to the statistics will show that in numerous instances, one or more of the viscera were greatly diseased; consequently, in those cases, the operation ought not to have been performed. The average proportion of such complications, was one in thirteen cases,—and where they existed, the mortality was thirteen in seventeen.

In all such cases, the operation is not to be justified; and we must charitably conclude that the operations in the cases referred to, could not have been aware of the complication, or they would not have undertaken them. The diagnosis was not accurately ascertained, and the results were fatal.

We have now, at the risk of reputation and prolixity, presented our views of the History, Diagnosis, and Pathology of ovarian tumors; and considered, also, at some length, the relative value of certain methods of treatment. The motives for this repetition and prolixity, will be better appreciated by the young practitioner, when he refers to the standard text books, and finds a few pages only devoted to so important a subject; and, especially, when he remembers that a few sentences from his medical teacher, comprises his whole education in the premises.

The different modes of operating in cystic dropsy, will next claim our attention.

There are two methods of performing ovariotomy, designated the Major and Minor operation. All operations, in which the incision varies from two and a half to six inches, are denominated minor, while where it exceeds six inches, the title of major operation is fully justified. These two modes of operating have divided surgeons into two classes,—those who favor the large incision, and those who prefer the small. The arguments of those holding to the major operation, appear very plausible, although the results presented by statistics are not so favorable as those arising from the minor section.

The advantages of the major operation, are said to be, that there is sufficient space for the operator to perform his manipulations; that the adhesions can be seen and cut through by the scalpel, instead of being torn by the hand: that the cyst can be removed entire from the abdomen, thus preventing the escape of fluid into its cavity—which circumstance is said to be a great source of mortality in the minor operation; that the fleshy masses connected with the cysts can be removed without difficulty, whereas, in the minor operation, they cannot be removed at all; and that if any blood or fluid escape into the cavity of the abdomen, it can be removed without injury, which is almost impossible in the minor operation. These are circumstances which the experienced operator can appreciate, and if he should not be blinded by undue fears of peritoneal inflammation, he will be sure to estimate highly such palpable advantages.

The objections raised to the major operation are, that in the majority of cases, the incision is unnecessarily long; that the same end can be attained by milder means; that from the extent of peritoneum exposed, there is more liability to inflammation; and that there is a greater liability to the escape of the intestines, and consequently a greater tendency for them to take on inflammatory action.

The principle of the minor incision, is to make as small an opening as possible through the parietes of the abdomen and peritoneum; sieze the sac with a volsellum, so that it should not recede when tapped; then puncture the cyst and evacuate the fluid; draw the sac through the opening, tie the pedicle, and detach it. This operation is admirably adapted to that form of the disease, in which the cyst is single, and uncomplicated with fleshy matter, and in a great many cases, this point can be pretty readily ascertained; but when adhesions to any extent exist, when the sac is multilocular, or when there is a large quantity of hardened substance, it would be impossible to withdraw the sac through a small opening.

Mr. Jefferson was the first person in England who adopted the small incision. In 1833, he operated successfully. The incision he used was about two and a half inches long; and after evacuating the fluid with a trocar, the sac was drawn through the incision and a ligature applied to the pedicle; the cyst was then removed. This mode of practice has been adopted by King, Lane, West, Philips, Bird, and many others.

On referring to the Tables we find, that of 85 patients where the large incision was employed, 50 were cured, and 35 died, making a mortality of *one* in *three* nearly; in 23 cases where the small incision was used, 19 were cured, and 3 died, making a mortality of nearly *one* in *six*. From this statement, therefore, we should arrive at the conclusion, that the small section is much more favorable to life than the large one.

We must remember, however, that many of the cases which were operated upon by the large incision, were quite unfit for the smaller operation; and, therefore, the patients would have been left to the natural course of disease, had not the major operation been performed. Again, a vast number of the cases presented the complication of strong adhesions, existing between the sac and parietes of the abdomen, which could not be overcome by Mr. Jefferson's mode of operation. And, lastly, we must bear in mind, that where the large section was used, the cases were more severe, and the complications greater, than in those of the other operation; from this cause alone there would of necessity be a greater mortality.

On the other hand, instances are recorded, where the boast of the operator has been the "entire" expulsion of the cyst, after the larger incision had been made, without adhesions or complications. *In such cases, would it not be better to employ a smaller section, and forego the triumph?*

But to conclude, if, after mature deliberation, and frequent examination, we are led to the conclusion, that the case under treatment, is one which presents a fair chance of success, if subjected to the operation, while, if it remains without surgical interference, the patient may die, the surgeon should not follow any particular plan laid down by his predecessors; but if the cyst can be extracted by the minor operation, it is the safest and best procedure. Again, if the minor section is commenced, and difficulties present themselves, nothing can be easier than the enlargement of the incision; thus giving the patient a chance of being cured by the safer operation, and, if it fail, we may still proceed to relieve her by the major operation.

Although it has been stated that the cyst should be removed entire, without rupture or tapping, we do not see very peculiar advantage to be derived from this procedure. It is true there may be danger of the fluid escaping into the abdominal cavity; but this accident will scarcely occur, if the cyst is carefully punctured, and due caution used in securing the opening by a ligature. We may then proceed to finish the operation.

III.—*What are the legitimate conclusions to be deduced, as to the practicability of the operation, upon a review of the subject?*

1. We have ascertained that ovarian dropsy is not so harmless a disease as some imagine; that, infact, under ordinary treatment, it is very fatal. More than half of the cases recorded, actually die—a large proportion of the others are reported only to be relieved—and only one in five recover.

2. That not only is it fatal, but that it is much more rapidly fatal than is generally supposed. The tables show that more than one-half, or sixty-three in 124, patients, die in less than two years; and more than half of these (viz, thirty-eight) died within twelve months from the commencement of the disease.

3. That tapping, which was formerly considered the only mode of palliating the disease, is a very dangerous remedy. In a table composed of thirty cases, fifteen died within four months after the operation, and twelve of the fifteen did not undergo a second tapping. In a table of forty-six cases, collected by Mr. Southam, twenty died after the first tapping. In a table of forty-six cases, cellected by Mr. Southam, twenty died after the first tapping. Sixteen of these cases died within one month of the operation, and ten of these sixteen died in seven days after the evacuation of the cyst.

4. Supposing the danger of the first tapping to have been escaped, we find that the fluid re-accumulates rapidly, and that the intervals between the operations become greatly diminished, while the quantity of fluid is increased, so that its remedial powers hardly compensate for the dangers which attend its performance.

5. We must remember that in some cases, paracentesis can be borne frequently, and life can be preserved in a tolerable state of comfort, for many years, under the careful performance of the operation. But these cases are rare, and should be regarded as exceptions to the rule.

6. That the operation of tapping ought only to be performed under one of two circumstances, namely, either early, when the cyst is unilocular, or when the tumor is producing serious pressure upon the vital organs. In no case, except under the latter circumstance, ought a multilocular cyst to be punctured, because the relief given is so trifling, and the dangers of paracentesis are so much increased, in this form of the disease.

7. That medical treatment produces only slight benefit. It may arrest the progress of the disease for some time, but very rarely effects a cure. Pressure as a remedy, prevents the cyst from enlarging rapidly.

8. That ovarian disease sometimes undergoes a spontaneous cure, by the rupture of the cyst, through the agency of ulceration, and the discharge of its contents into various outlets of the body.

9 That from the difficulty of curing the disease by the usual modes of treatment, the operation of the extraction of the cyst has been proposed, and performed nea.ly three hundred times, with average mortality of twenty-five per cent.

10. That in these three hundred operations, more than sixty, or about *one* in *five*, were unfinished, either from the extent of adhesions—from the tumor being uterine or omental—or from the fact that no tumor existed; proving, most indisputably, the great difficulty of correct diagnosis.

11. That the diagnosis is very obscure, as regards adhesions, and the character of the tumor; that adhesions existed in forty-six of eighty-one cases, and in six of the eighty-one there was no tumor.

12. That the mortality of the operation was greater where adhesions were present, than where they were absent.

13. That the disease is often complicated with organic disease of other viscera.

14. That the principal recorded *causes* of death, where it took place soon after the operation, are hemorrhageand peritonitis; but the cases are too few to be relied upon.

15. That when death takes place in consequence of the operation, it is very rapid. Of thirty patients, where time is mentioned, fourteen died within thirty-six hours, and twenty-five within a week.

16. That the character of the disease is of importance with regard to the mortality. In the extraction of hard tumors of the ovary, the mortality was more than fifty per cent.; while in those cases where the tumor was composed partly of fluid, and partly of solid matter, the mortality was less than thirty-three per cent. We conclude, therefore, that encysted tumors are much more favorable to the operation than hard or fibrous tumors.

17. That the mortality of the major operation is considerably greater than that of the minor section.

18. That in some cases the tumor is malignant; but that encysted dropsy is not, in the ordinary sense of the term, malignant, and that it may be removed without any tendency to malignant disease appearing subsequently in the pedicle.

Concluding Remarks.—We have now, so far as our limited researches will permit, placed the subject of Ovariotomy in its proper position; we have, without any bias for, or prejudice against the operation, exhibited the results as derived from reading and experience. We have endeavored to show that paracentesis and the ordinary modes of treatment, are unsatisfactory in their results, and only to be relied upon as palliative measures. And we have proved that Gastrotomy, or the excision of the cyst, affords the most rational ground of hope for a permanent cure; but whether this, or some other mode of treatment, will supersede the palliative measures, the general sentiment of the profession must decide. If a conscientious statement of principles and facts, will aid in bringing about this decision, we shall feel greatly rewarded for the labor bestowed upon this subject. Let those, whose position entitles them to give law to the profession, either allow that this operation is justifiable, or that it is not. If the results of its performance prove it to be legitimate, we may proudly boast the possession of another remedy for the alleviation of suffering humanity; but if not, then let all the powers of an enlightened profession be exerted to destroy this means of evil.

In the majority of cases which came under notice, it is our opinion, deliberately formed, that the operation is most decidedly unjustifiable. In encysted tumor, which has enlarged to such an extent as to demand active interference, or, when a unilocular cyst has beeen under treatment for some time, and is becoming multilocular by the addition of secondary or tertiary cysts upon its inner surface, then the operation is to be proposed and performed.

In such cases, if the diagnosis is clear; if it is believed that adhesions are absent, after the symptoms already pointed out have been intelligibly inquired of; and if the health of the patient is good, the surgeon is bound to extend to her the last aid of his art, and remove a tumor, which if allowed to remain, tends inevitably to her destruction. He should, however, first carefully and honestly, lay before the patient the dangers she must undergo; he should inspire her confidence by the relation of cases of successful extirpation; but he should, also, inform her of those less fortunate. He will thus acquire a confidence, which will be found very useful in the after-treatment, and upon which may depend the favorable result of the operation. If the cyst is single and uncomplicated with fibrous matter,—if the powers of life are active, and the spirits buoyant,—if the operator is skillful, and the after-treatment carefully attended to,—a successful issue may be anticipated.

Ovariotomy, however, is an operation which ought to be sought after. If it is to be the means of introducing surgeons into notice, it will be fearfully abused. We have known at least two operators, who devoted a large portion of their time tovisiting various parts of the country, seeking cases, and operating upon all, almost, indiscriminately. We have reason to believe that they are ignorant alike of the anatomical relations of the disease, and its diagnostic character; and yet their reprehensive adventures in surgery, have been blazoned in the village newspapers, and may ultimately find their way into the eastern medical journals. Such unprincipled charlatans should receive no "quarter" from the regular profession; their conduct deserves and should receive the unmitigated scorn of all honorable men.

The treatment after the operation is of great importance, as upon it very greatly depends the success of the operation. The plan of Dr. F. Bird—and he is one of the most successful operators—is a very simple but efficacious one. Its object is to place the skin in such a situation as to enable him at any time after the operation to cause profuse sweating. This is to be accomplished by elevating the temperature, and causing the patient to eat a considerable portion of ice; this at once produces free perspiration, and the patient is placed in comparative safety. If, however, pain in the abdomen comes on, the pulse becomes quick, and the moisture of the skin less; he again produces a higher temperature, giving ice, and continues to do so until the perspiration returns. The patient requires constant watching, and she ought not to be left for many days. The urine should be drawn off with the catheter, when not voided regularly; the bowels should be left undisturbed for several days, and then moved by enemata rather than by cathartic medicines. The diet must be restricted to toast, tea, rice and barley water, etc.

Before closing, we again urge that medical treatment should have a fair trial; for we should remember that this, and most other surgical operations, are the result of a deficiency in the *ars medicina*, and that the knife should be avoided in all cases where it is possible. That surgeon will be regarded as the greatest benefactor of the profession and the world, who will suggest some method by which the disease can be cured, without resorting to the terrible and hazardous operation of the excision of the cyst.—*Cincinnati Medical Observer.*

